# Variables associated with patient-reported outcomes and their trajectories following lower extremity free flap reconstruction

**DOI:** 10.1016/j.jpra.2026.07.030

**Published:** 2026-07-30

**Authors:** Mike Westmeijer, Emile B. List, Rutger M. Schols, Antonius J.M. Luijsterburg, E.S.J. van der Beek, J․ Henk Coert, Falco Hietbrink, Wiesje Maarse, Teun Teunis, David D. Krijgh, Dangle Research Group, Pien S.E. van Egdom, Arda Goceroglu, Nadine S. Hillberg, Jop Beugels, Marc A.M. Mureau, Shan S. Qiu, T.M.T. Tempelman

**Affiliations:** a Department of Plastic and Reconstructive Surgery, University Medical Center Utrecht, the Netherlands; b Department of Plastic and Reconstructive Surgery, Maastricht University Medical Center, Maastricht, the Netherlands; c Department of Plastic and Reconstructive Surgery, Erasmus MC, University Medical Center Rotterdam, Rotterdam, the Netherlands; d Department of Plastic and Reconstructive Surgery, University Medical Center Groningen, the Netherlands; e Department of Trauma Surgery, University Medical Center Utrecht, the Netherlands; f Department of Plastic Surgery, University of Pittsburgh Medical Center, USA

**Keywords:** Reconstruction, Free flap, Lower extremity, Long-term outcomes

## Abstract

**Background:**

Knowledge of potentially modifiable variables influencing the recovery and the trajectory of recovery after lower limb salvage with free flap reconstruction might aid in improving outcomes and support targeted rehabilitation strategies. Therefore, we asked: (1) what variables are independently associated with capability, quality of life, and discomfort, and their trajectory? (2) Is there a difference in recovery trajectories between specific capability tasks?

**Methods:**

Patients who were enrolled in the “Dangle study” and underwent lower extremity free flap reconstruction at four Dutch academic medical centers were invited to complete patient-reported outcome measures. Capability was assessed using PROMIS Physical Function – Mobility, quality of life using the EQ-5D-5 L, and discomfort using a numerical visual analogue scale (VAS). Questionnaires were completed at various times after surgery. Linear mixed-effects models were used to account for the repeated measures design.

**Results:**

Fifty-one participants completed 113 questionnaires. Male sex and the absence of diabetes were independently associated with greater capability, but not with recovery trajectories. Only time since reconstruction was independently associated with greater quality of life. Reconstructions of the foot were associated with increased levels of discomfort compared to lower leg reconstructions, but not with the trajectory of discomfort. Individual capability subscales demonstrated largely stable recovery trajectories without clinically meaningful differences between tasks.

**Conclusion:**

Although the variables identified are mostly non-modifiable, they can inform appropriate expectations before and after deciding on limb salvage. In addition, they can help identify people at greater risk for reduced recovery, who might benefit from targeted interventions such as the integrated involvement of rehabilitation, mental health, and pain management services.

## Introduction

Severe lower extremity wounds can leave vital structures such as bone, tendon or hardware exposed, thereby posing a complex reconstructive challenge. When local tissue cannot provide adequate soft tissue coverage, these injuries benefit from a free tissue transplantation.^1^, ^2^, ^3^ Due to surgical advances, free flap reconstruction has become a common alternative to limb amputation.^4^, ^5^, ^6^

Although limb reconstruction has not been consistently associated with superior capability compared to amputation, limb salvage may confer psychological benefits.^7^, ^8^, ^9^ Regardless, capability and mental health remain reduced following reconstruction, and ongoing discomfort is common, contributing to delayed return to work, reduced productivity, and increased healthcare utilization.^9^, ^10^, ^11^, ^12^, ^13^, ^14^, ^15^ Several studies have identified patient- and reconstruction related variables associated with postoperative outcomes.^10^^,^^15^^,^^16^ However, these studies do not explore recovery trajectories. Knowledge of potentially modifiable variables influencing the recovery and the trajectory of recovery might aid in improving outcomes. In addition, knowledge of longitudinal changes in specific capability tasks may support targeted rehabilitation strategies to improve recovery of capability.

Therefore, we asked: (1) what variables are independently associated with capability, quality of life, and discomfort, and their trajectory, in the years following lower extremity free flap reconstruction? (2) Is there a difference in recovery trajectories between specific capability tasks?

## Methods

### Study design and participants

This multicenter, prospective cohort study examined long-term capability, quality of life, and discomfort following lower extremity free flap reconstruction. All patients who were initially enrolled in the “Dangle study” agreed to post-reconstructive follow-up and were included in this study.^17^ Inclusion and exclusion criteria of the “Dangle study” are presented in Table 1.

**Table 1:** Inclusion and exclusion criteria of the “Dangle study”.

**Inclusion criteria**	- Male or Female - Aged between 16–99y old - Free flap reconstruction of the lower leg

**Exclusion criteria**	- Insufficient Dutch proficiency - Patients unable to give informed consent - Patients who were mentally incapacitated - Reconstruction requiring ≥2 free flaps - Patients who were undergoing a secondary free flap reconstruction due to partial or total free flap necrosis

### Data collection and measurement tools

Patient baseline characteristics were extracted from the “Dangle study” database, and included demographics, defect location and etiology, and the type of free flap used for reconstruction. Capability was measured through the Dutch Patient-Reported Outcomes Measurement System (PROMIS) v2.0 Physical Function – Mobility.^18^ Quality of life and discomfort were assessed using the five-level EuroQol-5 Dimension (EQ-5D-5 L) and the numerical Visual Analogue Score (VAS), respectively.^19^^,^^20^ All patients were regularly invited to fill out a set of these questionnaires. Because follow-up schedules were patient-specific, questionnaires were completed at non-fixed time points. Questionnaire data was aggregated and stored in Castor EDC (Amsterdam, The Netherlands).

PROMIS Physical Function – Mobility scores were presented as T-scores, which as standardized to the general population (mean 50, SD 10). T-scores above 50 therefore reflect better than average function, while scores below 50 represent reduced mobility.^21^ Our instrument consisted of fourteen individual subscales assessing different aspects of lower extremity capability. Examples of items included the ability to walk at a normal speed, climb several flights of stairs, and jump up and down. Each subscale was scored on a 5-point Likert scale ranging from 1 (“unable to do”) to 5 (“without any difficulty”). EQ-5D-5 L scores ranged from zero (i.e., “the worst health you can imagine”) to one hundred (i.e., “the best health you can imagine”). The EQ-5D-5 L comprises five dimensions: mobility, self-care, usual activities, pain/discomfort, and anxiety/depression. VAS pain was measured using a numerical scale ranging from 0–100, with higher scores indicating greater pain intensity over the seven days preceding the assessment.

### Statistical analysis

Patient baseline characteristics were presented as numbers with percentages (%), mean with standard deviation (SD), or median with interquartile range (IQR). We used multivariable linear mixed-effects models to identify variables independently associated with capability, quality of life, and discomfort, and their trajectories, while accounting for the study’s repeated measures design. Variables with P-values < 0.10 in bivariate mixed-effects analysis were entered into the final multivariable models (Supplementary Table 1–3). Time was included into all models as an independent variable to account for within-patient longitudinal variation. Interaction terms between time and significant independent variables were created to assess the association with recovery trajectories. To determine differences in recovery trajectories between specific capability tasks, we performed bivariate linear mixed-effects models for each capability subscale accounting for time. Regression coefficients with non-overlapping 95% confidence intervals were regarded as different recovery trajectories. Statistical analyses were performed using R version 4.4.2. We had no missing data.

## Results

### Patient characteristics

Fifty-one participants (64%, total n = 80) completed all questionnaires at least once. Each questionnaire was completed 113 times. Participants mean age was 55 (SD = 17) years, and 69% (n = 35) of respondents were male. Three-quarter of all wounds affected the lower leg, and about two-thirds were trauma related. Reconstructions were performed using both muscle flaps (45%, n = 23) and fasciocutaneous flaps (55%, n = 28) (Table 2).

**Table 2:** Patient baseline characteristics.

Patients, n	51
Response proportion (%) a	64%
Number of completed questionnaires	113
Male sex, n (%)	35 (69%)
Age in years, mean (SD)	55 (17)
**Comorbidities, n (%)**	
Smoking	7 (14%)
Hypertension	12 (24%)
Diabetes mellitus	3 (5.9%)
Cardiovascular disease	12 (24%)
**Lower extremity defect, n (%)**	
**Location**	
Lower leg	38 (75%)
Foot	13 (26%)
**Cause of Defect**	
Trauma	32 (63%)
Others b	19 (37%)
**Gustilo classification**^c^	
3A	6 (19%)
3B	13 (41%)
3C	2 (6.3%)
Other	11 (34%)
**Type of flap, n (%)**	
Muscle	23 (45%)
Fasciocutaneous	28 (55%)

a Questionnaires were send to all participants of the “Dangle Study” (N = 80).

b Others included defects due to oncological resection, chronic infection or osteomyelitis, postoperative complications (e.g. wound dehiscence, non-union, hardware exposure), pressure ulcers, and (chemical) burns.

c As part of the number of trauma patients.

Median PROMIS Physical Function – Mobility T-scores ranged between 34 and 39 in the three years following surgery. Median EQ-5D-5 L scores ranged from 62 to 85, and median VAS pain scores ranged from 21 to 71 (Supplementary Table 4).

### Variables independently associated with capability, quality of life, and discomfort and their trajectory

Male sex (*β =* 4.6, 95% CI 0.19 to 9.1, p = 0.041) and the absence of diabetes (*β* = −13, 95% CI-22 to −3.9, *p* < .01) were independently associated with greater capability, but not with the recovery trajectory. Time since reconstruction was not associated with capability.

Time since reconstruction was the only variable that could be modeled for quality of life due to singular covariance structures reflecting the limited within-patient variability. This occurred because EQ-5D-5 L scores showed minimal change within individual patients over time, making it statistically unreliable to estimate the independent effects of additional covariates. Time since reconstruction was independently associated with greater quality of life (*β* = 0.20, 95% CI 0.030 to 0.37, p = 0.021).

Lower leg location of the wound (*β* = −16, 95% CI −32 to −0.62, p = 0.042) was independently associated with reduced levels of discomfort, but not with the trajectory of discomfort. Time since reconstruction was not associated with discomfort (Table 3).

**Table 3:** Multivariable linear mixed-effects models.

	ß coefficient	95% CI	P-value
**Capability**			
Male sex	4.6	(0.19 to 9.1)	**0.041**
Diabetes mellitus	−13	(−22 to −3.9)	**0.0050**
Time in Months	−0.060	(−0.14 to −0.020)	0.12
Time * Diabetes mellitus	0.058	(−0.30 to 0.41)	0.75
Time * Male sex	0.052	(−0.12 to 0.22)	0.55
**Quality of Life**			
Time in Months	0.20	(0.030 to 0.37)	**0.021**
**Discomfort**			
Lower leg location	−16	(−32 to −0.62)	**0.042**
Muscle flap	−12	(−25 to 1.8)	0.091
Time in Months	−0.080	(−0.30 to 0.14)	0.47
Time * Lower leg location	−0.23	(−0.75 to 0.29)	0.39

### Differences in trajectories between specific capability tasks

Time since reconstruction was positively associated with the ability to jump up and down (*β* = 0.016, 95% CI 0.0020 to 0.030, p = 0.023), and negatively associated with the ability to go for a short walk (*β* = −0.014, 95% CI −0.027 to −0.00020, p = 0.049), and climb several flights of stairs (*β* = −0.012, 95% CI −0.023 to −0.00090, p = 0.037). No significant associations were observed for the remaining subscales (Supplementary Table 5).

## Discussion

Despite successful limb salvage, patients continue to experience reduced capability following free tissue transplantation, contributing to delayed return to work, reduced productivity, and increased healthcare utilization.^9^, ^10^, ^11^, ^12^, ^13^, ^14^, ^15^ Knowledge of potentially modifiable variables influencing recovery, or the trajectory of recovery, might allow targeted interventions aimed at optimizing outcomes. Male sex and the absence of diabetes were associated with greater capability, while reconstructions of the foot were associated with increased discomfort. There were no variables independently associated with recovery trajectories. Moreover, individual capability tasks demonstrated little to no improvement over time. Although generally non-modifiable, knowledge of the variables associated with outcomes can improve setting appropriate expectations before and after deciding on limb salvage and identify people at greater risk for reduced recovery, who might benefit from targeted interventions such as the integrated involvement of rehabilitation, mental health, and pain management services.

### Limitations

This study has several limitations. First, the number of responses is somewhat modest, particularly earlier after free flap application. This is likely due to less frequent reminders for questionnaire completion during the early period after surgery. As a result, associations between low-prevalence variables and recovery trajectories may have been underpowered and therefore remain undetected. However, the modest number of responses does not invalidate our findings as linear mixed-effects models are robust to unbalanced longitudinal data. Second, not all patients completed multiple questionnaires, which typically complicates individual change estimates over time. This was addressed by using linear mixed-effects models, which appropriately account for repeated measures and varying numbers of observations per patient. Third, the prospective cohort design exhibits the risk of selection and attrition bias. Patients who were enrolled in this study and completed postoperative follow-up may differ from those who were not enrolled or were loss to follow-up. We compared baseline characteristics of patients with and without questionnaires and found no significant difference (Supplementary Table 6). Fourth, we did not include important measures of mental and social health associated with capability in prior studies.^22^ Future studies can include such variables to further improve prognostication.

### Variables independently associated with capability, quality of life, and discomfort and their trajectory

Male sex and the absence of diabetes were independently associated with greater capability, but not with capability trajectories. Notably, time since reconstruction was not associated with capability, underscoring the persistent reduced capability despite successful limb salvage. This aligns with previous research demonstrating poor long-term capability following lower extremity free flap reconstruction.^9^, ^10^, ^11^ Patients with diabetes mellitus reported capability scores more than one standard deviation below those without diabetes mellitus, which may reflect the known effects of microvascular disease, peripheral neuropathy, and reduced exercise tolerance, as well as worse postsurgical outcomes seen in patients with diabetes.^16^^,^^23^, ^24^, ^25^, ^26^^,^^16^^,^^24^^,^^25^ This knowledge can help physicians set appropriate patient expectations regarding capability after free flap reconstruction.

Time since reconstruction was positively associated with quality of life, although the effect size was small and its clinical relevance seems limited. Regardless, it stems hopeful that the expected reduced quality of life after major reconstructive surgery can recover for some time. Moreover, EQ-5D-5 L scores were notably lower than those reported in Western European populations.^27^, ^28^, ^29^, ^30^ Our findings underscore the long-lasting impact of these injuries.

Foot reconstructions were associated with higher levels of discomfort compared to lower leg reconstructions, likely reflecting the biomechanical and weightbearing challenges unique to the foot region.^31^ However, defect location was not associated with recovery of discomfort.

More importantly, discomfort did not improve over time. This is consistent with previous studies that indicated that discomfort persists following lower extremity trauma and free flap reconstruction.^11^^,^^32^ Several studies have shown that improved mental and social health are associated with greater capability and reduced discomfort.^22^^,^^33^^,^^34^ Moreover, evidence suggests that psychological interventions, particularly cognitive behavioral therapy, may improve psychological well-being following traumatic injury.^35^ Future studies can test if integrating psychological interventions following lower extremity free flap reconstruction can improve capability and reduce discomfort.

### Differences in trajectories between specific capability tasks

Most individual capability subscales demonstrated little to no improvement over time following reconstruction. This is important, as it indicates that early postoperative capability is predictive of long-term outcomes, suggesting limited additional benefit from prolonged rehabilitation. Healthcare providers can use this information to set more realistic expectations regarding recovery. By examining trajectories of individual capability tasks rather than overall capability, this study provides additional insight into how different aspects of physical function evolve over time and may help inform future rehabilitation strategies.

## Conclusion

Findings from this study underscore that, despite successful limb salvage, patients continue to experience reduced capability, quality of life, and increased discomfort. The variables described in this study can help identify people with a greater probability of reduced incapability, quality of life and increased discomfort. Targeted multidisciplinary and psychosocial interventions may be beneficial in optimizing long-term outcomes, although our findings question the benefit of prolonged rehabilitation. Furthermore, knowledge of variables associated with outcomes may support preoperative counseling and expectation management in patients undergoing lower extremity free flap reconstruction.

## Ethics approval and informed consent

The study was approved by the METC (Dutch Medical Research Ethics Commission) in the Netherlands on 11 July 2018: NL63146.041.17 and registered with the Dutch Trial Register (registration number NTR 7545). The handling of personal data was performed in compliance with the Dutch Personal Data Protection Act and in compliance with Good Clinical Practice guidelines. Informed consent forms from all study participants were recorded at every hospital in the electronic patient file and signed paper forms were securely locked up within the hospital where the patient underwent treatment. The study was monitored by an independent monitoring company (Julius Clinical, Zeist, The Netherlands) according to a detailed monitoring plan.

## Declaration of competing interest

No funding was received for this study. None of the authors have a conflict of interest.

## Appendix Supplementary materials

Image, application 1
