# Supplementary material for: Variables associated with patient-reported outcomes and their trajectories following lower extremity free flap reconstruction
Source: JPRAS Open. 2026 Jul 30;51:742–9. doi: 10.1016/j.jpra.2026.07.030 (PMC13520620; doi:10.1016/j.jpra.2026.07.030)
Supplement: Supplementary file 1 [file mmc1.docx]

Supplemental

**Variables Associated with Patient-Reported Outcomes and Their Trajectories Following Lower Extremity Free Flap Reconstruction**

Mike Westmeijer M.D.^1^

Emile B. List M.D.^1^

Rutger M. Schols, M.D., Ph.D.^2^

Antonius J.M. Luijsterburg, M.D., Ph.D.^3^

E.S.J. van der Beek, M.D., Ph.D.^4^

J. Henk Coert M.D., Ph.D.^1^

Falco Hietbrink, M.D., Ph.D.^5^

Wiesje Maarse M.D., Ph.D.^1^

Teun Teunis, M.D., Ph.D.^6^

David D. Krijgh M.D., Ph.D.^1^

Dangle Research Group (Pien S.E. van Egdom, M.D.; Arda Goceroglu, M.D., Ph.D; Nadine S. Hillberg, M.D., Ph.D; Jop Beugels, M.D., Ph.D; Marc A.M. Mureau, M.D., Ph.D.; Shan S. Qiu, M.D., Ph.D.; T.M.T. Tempelman, M.D.)

1. *Department of Plastic and Reconstructive Surgery, University Medical Center Utrecht, the Netherlands*
2. *Department of Plastic and Reconstructive Surgery, Maastricht University Medical Center, Maastricht, the Netherlands*
3. *Department of Plastic and Reconstructive Surgery, Erasmus MC, University Medical Center Rotterdam, Rotterdam, the Netherlands*
4. *Department of Plastic and Reconstructive Surgery, University Medical Center Groningen, the Netherlands*
5. *Department of Trauma Surgery, University Medical Center Utrecht, the Netherlands*
6. *Department of Plastic Surgery, University of Pittsburgh Medical Center, USA*

Corresponding Author:

Mike Westmeijer, M.D.
Department of Plastic and Reconstructive Surgery
University Medical Center Utrecht, Heidelberglaan 100, 3508 GA Utrecht, The Netherlands

[m.westmeijer-3@umcutrecht.nl](mailto:m.westmeijer@students.uu.nl)

The authors have no conflict of interest or financial disclosures

**Ethical statement:** This study is in accordance with the 1964 Declaration of Helsinki and was approved by the CCMO Dutch Medical Research Ethics Commission.

Supplementary Table 1. Capability: Bivariate Multivariable Linear Mixed-Effects Model

| **Variable** | **P-value** |
| --- | --- |
| Age | 0.10 |
| Male sex | **0.069** |
| Smoking | 0.11 |
| Hypertension | 0.22 |
| Diabetes mellitus | **0.0098** |
| Cardiovascular disease | 0.76 |
| Traumatic cause | 0.93 |
| Lower leg location | 0.56 |
| Muscle Flap | 0.45 |

Note: male sex (p = 0.069), and diabetes mellitus (p = 0.0098) met the p < 0.10 threshold for inclusion in the multivariable model.

Supplementary Table 2. Quality of Life: Bivariate Multivariable Linear Mixed-Effects Model

| **Variable** | **P-value** |
| --- | --- |
| Time | 0.021 |

Note: only the time since reconstruction was associated (p = 0.021), and included in the multivariable model. All other variables resulted in non-estimable models due to singular covariance structures.

Supplementary Table 3. Discomfort: Bivariate Multivariable Linear Mixed-Effects Model

| **Variable** | **P-value** |
| --- | --- |
| Age | 0.74 |
| Male sex | 0.12 |
| Smoking | 0.12 |
| Hypertension | 0.89 |
| Diabetes mellitus | 0.94 |
| Cardiovascular disease | 0.29 |
| Traumatic cause | 0.34 |
| Lower leg location | **0.021** |
| Muscle Flap | **0.044** |

Note: lower leg location (p = 0.021), and the use of muscle flaps (p = 0.044) were included in the final multivariable model.

Supplementary Table 4. Median PROMIS, EQ-5D-5L, and VAS Pain Scores

| **Time Interval (Months)** | **Median PROMIS  T-score (IQR)** | **Median EQ-5D-5L score (IQR)** | **Median VAS Pain score (IQR)** |
| --- | --- | --- | --- |
| 0 to 6 | 37 (12) | 65 (24) | 37(26) |
| 6 to 12 | 37 (11) | 62 (18) | 27 (38) |
| 12 to 18 | 39 (11) | 70 (29) | 40 (42) |
| 18 to 24 | 37 (12) | 70 (19) | 21 (57) |
| 24 to 30 | 37 (11) | 70 (33) | 28 (35) |
| 30 to 36 | 34 (11) | 85 (38) | 71 (38) |
| 36+ | 36 (13) | 83 (30) | 28 (42) |

Supplementary Table 5. Capability Subscales: Bivariate Multivariable Linear Mixed-Effects Models

| **Subscale of Capability** | 𝞫 **coefficient of Time** | **95% CI** | **P-value** |
| --- | --- | --- | --- |
| Q1. Ability to walk at normal speed *^a^* | -0.0064 | (-0.020 to 0.0073) | 0.36 |
| Q2. Ability to stand up from an armless straight chair *^a^* | -0.0072 | (-0.018 to 0.0033) | 0.18 |
| Q3. Ability to go up and down the stairs at a normal pace *^a^* | 0.0019 | (-0.0090 to 0.013) | 0.73 |
| Q4. Ability to walk for at least fifteen minutes *^a^* | -0.014 | (-0.029 to 0.0017) | 0.084 |
| Q5. Ability to get up from the floor from lying on your back without help *^a^* | -0.0060 | (-0.019 to 0.0073) | 0.38 |
| Q6. Ability to jump up and down *^a^* | 0.016 | (0.0020 to 0.030) | **0.023** |
| Q7. Ability to climb up five steps *^a^* | -0.0043 | (-0.017 to 0.0086) | 0.51 |
| Q8. Ability to run a short distance (e.g. to catch a bus) *^a^* | -0.0016 | (-0.010 to 0.0069) | 0.72 |
| Q9. Ability to stand unsupported for ten minutes *^a^* | -0.014 | (-0.028 to 0.0013) | 0.077 |
| Q10. Ability to stand for one hour *^a^* | -0.010 | (-0.024 to 0.0037) | 0.15 |
| Q11. Ability to stand unsupported for thirty minutes *^a^* | -0.013 | (-0.026 to 0.00070) | 0.065 |
| Q12. Limited in climbing one flight of stairs *^b^* | -0.0034 | (-0.015 to 0.0081) | 0.56 |
| Q13. Limited in going for a short walk (less than fifteen minutes) *^b^* | -0.014 | (-0.027 to -0.00020) | **0.049** |
| Q14. Limited in climbing several flights of stairs *^b^* | -0.012 | (-0.023 to -0.00090) | **0.037** |

^a^ Each question was scored as “without any difficulty,” (score 5) “with a little difficulty,” (score 4) “with some difficulty,” (score 3) “with much difficulty,” (score 2) and “unable to do” (score 1).

^b^ Each question was scored as “not at all,” (score 5) “very little,” (score 4) “somewhat,” (score 3) “quite a lot,” (score 2) and “cannot do” (score 1).

Supplementary Table 6. Patient Baseline Characteristics of Questionnaire Responders and Non-Responders

|  | **Responders (N=51)** | **Non-responders (N=29)** | **P-value *^a^*** |
| --- | --- | --- | --- |
| Male sex, n (%) | 35 (69%) | 17 (58%) | 0.47 |
| Age in years, mean (SD) | 55 (17) | 60 (15) | 0.18 |
| **Comorbidities, n (%)** |  |  |  |
| Smoking | 7 (14%) | 4 (14%) | 1.00 |
| Hypertension | 12 (24%) | 4 (14%) | 0.39 |
| Diabetes mellitus | 3 (5.9%) | 6 (21%) | 0.065 |
| Cardiovascular disease | 12 (24%) | 4 (14%) | 0.39 |
| **Lower extremity defect, n (%)** |  |  |  |
| **Location** |  |  | 0.79 |
| Lower leg | 38 (75%) | 23 (79%) | - |
| Foot | 13 (26%) | 6 (21%) | - |
| **Cause of Defect** |  |  | 0.81 |
| Trauma | 32 (63%) | 17 (59%) | - |
| Others *^b^* | 19 (37%) | 12 (41%) | - |
| **Gustilo classification *^c^*** |  |  | 0.92 |
| 3A | 6 (19%) | 2 (12%) | - |
| 3B | 13 (41%) | 7 (41%) | - |
| 3C | 2 (6.3%) | 1 (5.9%) | - |
| Other | 11 (34%) | 7 (41%) | - |
| **Type of flap, n (%)** |  |  | 0.64 |
| Muscle | 23 (45%) | 15 (52%) | - |
| Fasciocutaneous | 28 (55%) | 14 (48%) | - |

*^a^* Continuous variables were compared using independent samples t-tests and categorical variables using Fisher’s exact test *^b^* Others included defects due to oncological resection, chronic infection or osteomyelitis, postoperative complications (e.g. wound dehiscence, non-union, hardware exposure), pressure ulcers, and (chemical) burns.

*^c^* As part of the number of trauma patients.
